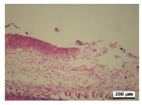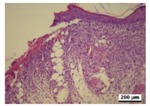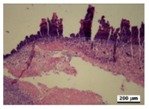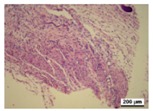# Biomedical Potential of Ultrafine Ag Nanoparticles Coated on Poly (Gamma-Glutamic Acid) Hydrogel with Special Reference to Wound Healing

**DOI:** 10.3390/nano8050324

**Published:** 2018-05-14

**Authors:** Yu Wang, Chunyan Dou, Guidong He, Litong Ban, Liang Huang, Zheng Li, Jixian Gong, Jianfei Zhang, Peng Yu

**Affiliations:** 1 College of Biotechnology, Tianjin University of Science and Technology, Tianjin 300457, China; wytjac@163.com; 2 College of Agronomy and Resources Environment, Tianjin Agricultural University, Tianjin 300384, China; banlitong@126.com (L.B.); huangliang@tjau.edu.cn (L.H.); 3 Key Laboratory of Advanced Textile Composites, Ministry of Education; School of Textiles, Tianjin Polytechnic University, Tianjin 300387, China; 15620671751@163.com(C.D.); calvinheguidong@163.com (G.H.); gongjixian@163.com (J.G.); zhangjianfei1960@126.com (J.Z.)

**Keywords:** biomedical, Ag nanoparticles, poly (gamma-glutamic acid) hydrogel, wound healing

## Abstract

In wound care management, the prevention of wound infection and the retention of an appropriate level of moisture are two major challenges. Therefore, designing an excellent antibacterial hydrogel with a suitable water-adsorbing capacity is very important to improve the development of wound dressings. In this paper, a novel silver nanoparticles/poly (gamma-glutamic acid) (γ-PGA) composite dressing was prepared for biomedical applications. The promoted wound-healing ability of the hydrogels were systematically evaluated with the aim of attaining a novel and effective wound dressing. A diffusion study showed that hydrogels can continuously release antibacterial factors (Ag). Hydrogels contain a high percentage of water, providing an ideal moist environment for tissue regeneration, while also preventing contraction of the wound. Moreover, an in vivo, wound-healing model evaluation of artificial wounds in mice indicated that silver/γ-PGA hydrogels could significantly promote wound healing. Histological examination revealed that hydrogels can successfully help to reconstruct intact epidermis and collagen deposition during 14 days of impaired wound healing. Overall, this research could shed new light on the design of antibacterial silver/γ-PGA hydrogels with potential applications in wound dressing.

## 1. Introduction

Skin is an indispensable natural barrier organ that protects internal organs from external environmental infection [1]. If skin is damaged, it will lose its protective function. Thus, microorganisms that enter the body can easily invade and begin to breed. Therefore, this can lead to wound infection [2] and even life-threatening illnesses [3]. However, wound care management is very difficult because infections may lead to exudate formation, which can lead to delayed wound healing and improper collagen deposition [4,5]. Microorganisms, such as *Staphylococcus aureus* and *Escherichia coli* [6], are a major cause of infection. In addition, serious wound dehydration can disturb the moist healing environment and delay wound healing [7]. Therefore, many efforts have been made to develop different materials to protect damaged skin.

An ideal wound dressing should be nontoxic, nonallergenic, nonadherentto skin, and able to absorb excess exudates to maintain an ideal moist environment for the wound [4]. Further, an ideal would dressing should possess biocompatible and antimicrobial properties to enhance wound healing, as well as a self-cleaning ability to prevent contamination [8,9].

To achieve this goal, researchers have developed different kinds of wet dressings. In recent years, chitosan [10], γ-poly(glutamic acid) [11,12], gelatin [13,14] and bacterial cellulose [15,16,17] have been widely used as hydrogel wound dressings because of their non-toxic, biocompatible, moisture retentive, readily-available and biodegradable properties. Hydrogels are three-dimensional hydrophilic polymeric networks that can absorb and retain a large amount of water [6], which also prevents contraction of a wound. An ideal moist environment improves the supply of white blood cells, enzymes, cytokines, and growth factors [18]. Unfortunately, the moist environment could also benefit the growth of microorganisms. Therefore, the design of hydrogels with a synergistic antimicrobial agent is a significant goal in the wound dressing industry. Hydrogels, which can be flakes, pastes, or gel fibers, provide a large free space between the crosslinked networks in the swollen stage. The free space could be a nano-reactor for the growth and/or nucleation of nanoparticles [6]. It is well-known that silver-based materials are highly effective againstmicro-organisms [19,20]. Nano-Ag particles and their mechanisms of inhibition have been demonstrated to be due to their ability to destabilize the outer membrane, collapse the plasma membrane potential, and deplete the levels of intracellular ATP of *E. coli* [19]. Therefore, Ag/hydrogel has been widely studied in wound healing [21], however, the aggregation of Ag nanoparticles may restrict antimicrobial activity due to the decrease in surface activation energy. In order to overcome this restriction, Ag nanoparticles (AgNpP) can be mixed with other polymer materials, such as poly (l-lactide-co-glycolide) [22,23], polymethacrylic acid [24], poly(acrylic acid) [25], poly(ethylene glycol) [26], poly(vinyl alcohol) [27], pectin [28], chitosan [29], and carboxymethyl cellulose [30]. Moreover, a controlled Ag nano-cluster deposition into polyvinyl alcohol/gum acacia (PVA-GA) hydrogel has already been achieved [31]. Abdelgawad et al. [32] developed a multicomponent (chitosan/silver-NPS/polyvinyl alcohol) system via electrospinning and studied its antimicrobial activity. Stevanovic et al. [33] reported the synthesis of microgels consisting of poly(l-glutamic acid)-capped AgNpP. An approach for the stabilization of AgNpP in-graphene hydrogel was also studied by crosslinking with acrylic acid and *N*,*N*′-methylene bisacrylamide [34]. In this work, we designed a novel AgNpP with a poly(γ-glutamic acid) hydrogel, which could offer an accelerated wound regeneration environment with the release of the AgNpP antimicrobial factor.

## 2. Materials and Methods

### 2.1. Materials

Poly(γ-glutamic acid) (γ-PGA; average molecular weight, 730 KDa) was purchased from Shineking Biotechnology (Nanjing, China). Ethylene glycol diglycidyl ether (EGDE) was purchased from Tongshi Chemical Ltd. (Yantai, China). Nano silver solution (2000 μg/mL) was purchased from Luzheng Nano Technology Co., Ltd. (Shanghai, China). The zeta potential of the nanoparticles was 18.63 ± 0.57 mv and the size distribution was 44.8 ± 10.9 nm.

### 2.2. Methods

#### 2.2.1. Synthesis of Ag-Hydrogel Copolymer

The Ag-hydrogel copolymer was synthesized according to our previously published method, following a chemical method from the monomers γ-PGA and EGDE at a mass ratio of 100:40 [35]. Before cross-linking, different concentrations of nano silver solution were added drop-wise into the γ-PGA mixture. The Ag-(γ-PGA) hydrogel copolymer was analyzed using UV-vis spectrophotometry to confirm the absorbance of silver nanoparticles.

#### 2.2.2. Preparation of Hydrogel Samples

**Figure 1:**
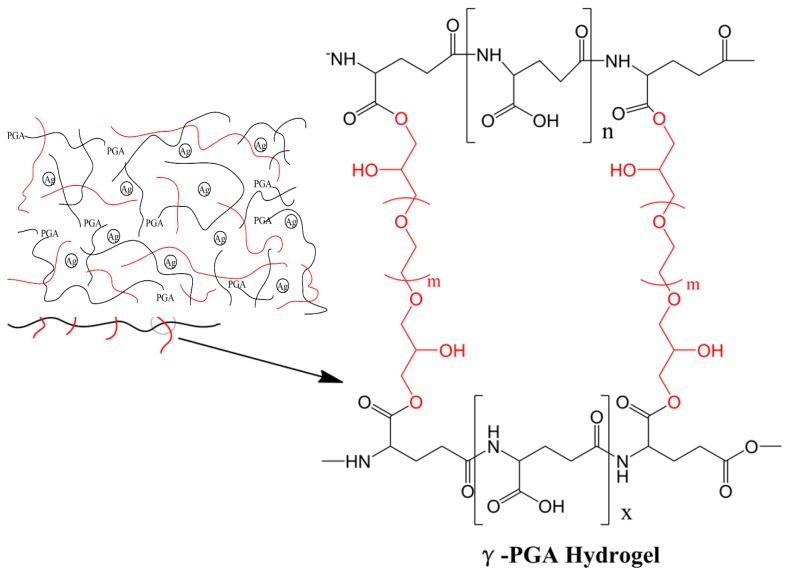
Hydrogel preparation by cross-linking of the carboxyl groups on the polymer chains using anethylene glycol diglycidyl ether. Poly(γ-glutamic acid) and ethylene glycol diglycidyl ether are represented by black and red interconnected lines, while the chemical structure at the cross-link site is illustrated. Ag particles are loaded in crosslinked hydrogels. Prior to the addition of Ag particles to synthesize the Ag-hydrogel copolymer, the copolymer was mixed with EGDE as an aqueous solution.

A 12 wt % γ-PGA copolymer solution was prepared using deionized water. Then, 75 mL of this polymer solution was transferred into separated glass beakers. Ag was measured by the concentration in its solution and was added to these beakers to concentrations of 0.0, 5.0, and 20.0 μg/mL. A total of 4.0 g of EGDE was added to each of the beakers in order to crosslink and make hydrogels. The pH of the solutions were adjusted to 4.0 by a pH meter at 50 °C for 20 h, as shown in Figure 1. The mixture formed film-shaped samples due to the cross-linking. All experiments were conducted in duplicate.

#### 2.2.3. Water Absorption Capacity of the Hydrogel

The hydrogel films were suspended in glass beakers containing 200 mL Britton–Robinson buffer solution (pH = 4.0–8.0) at room temperature. At a suitable time, the films were taken out and immediately weighed, after the excess water was removed carefully with bibulous paper. Measurements were performed twice [36].

#### 2.2.4. Diffusion Study

A simple release assay was conducted to study the release of AgNpP over a predetermined time in a PBS buffer solution, which can be used to indirectly evaluate antibacterial ability. First, the wavelength of AgNpP was investigated using an ultraviolet and visible spectrophotometer (Hach DR6000) (200–700 nm). Then, the release study was conducted on the hydrogels containing AgNpP, that were prepared twice (*n* = 2), as mentioned earlier, at aconcentration of 5.0 μg/mL. The hydrogel samples were soaked in 50 mL of distilled water at 37 °C. The release study was conducted over 48 h; after a given time, the concentration of Ag was measured using an ultraviolet and visible spectrophotometer.

#### 2.2.5. In Vivo Study

A total of 60 male Balb/C mice, 20–25 g in weight and 8 weeks old, were obtained from the Experimental Animal Center at the Academy of Military Medical Sciences. Mice were housed, one per cage, and all mice were fed a standard diet and water, under a normal standardized temperature and a relative humidity range of 50% to 60%. The mice were acclimatized for one week prior to experiments and were excluded if they lost weight during this period. On the day of wounding, the mice were anaesthetized with pentobarbital sodium and dorsal hairs were clipped; full thickness-round wounds (about 100 nm) were created on the upper back of each mouseusing a sharp pair of scissors and a scalpel. The prepared wounds in the four groups were tightly covered with γ-PGA, γ-PGA/Ag-5, and γ-PGA/Ag-20, respectively. Wounds with gauze dressing were kept as a negative control. The dressing materials were changed every two days. During the changing of the dressings, the area of the wound was measured using the paper weighing method and photographs were taken. The wound healing ratio was calculated using the following equation:

$$\mathrm{wound}\;\mathrm{healing}\;\mathrm{ratio}=\frac{A_{0}-A_{t}}{A_{0}}\times 100\%$$

where *A*_0_ is the wound area at the day the wound was created and *A_t_* is the wound area at the day of the changing of the dressing.

On day 4, 7, 10, and 14 after surgical treatment, the wounds were photographed for the measurement of wound size reduction. Then, for histological examination, the skin tissue samples, including the entire wound with adjacent normal skin, were excised at the above-mentioned time and fixed in 10% formalin for more than 24 h before being dehydrated. Then, the excised wound samples fixed in formalin were processed and embedded inparaffin. The serial sections, 3–5 μm thick, of tissue were stained with hematoxylin and eosin (H&E). The samples were observed for morphological changes using an optical microscope (Olympus CKX41, Leica, Tokyo, Japan).

## 3. Results and Discussion

### 3.1. Synthesis of Hydrogel

**Figure 2:**
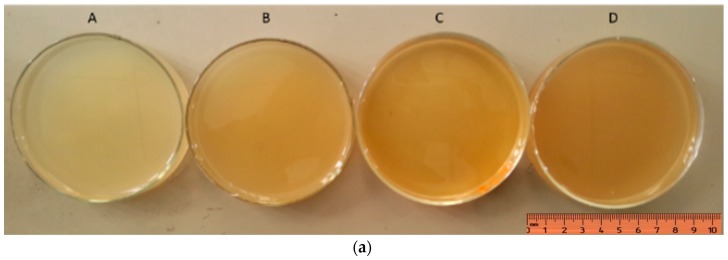

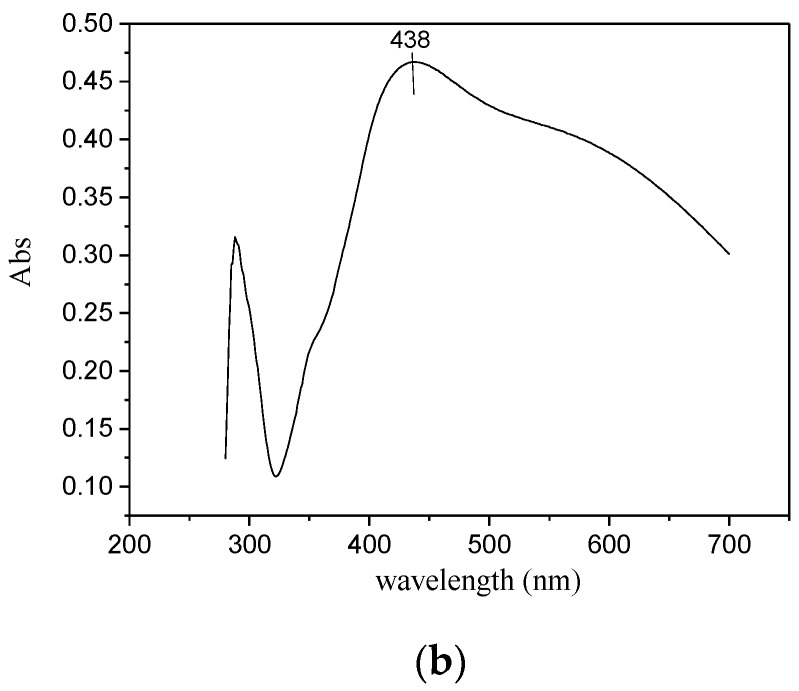
(**a**) Hydrogel samples (the concentration of Ag particles, A: 0.0 μg/mL; B: 5.0 μg/mL; C: 10.0 μg/mL; D: 20.0 μg/mL); (**b**) UV-visible spectrum of nano-Ag.

Hydrogel samples were synthesized using the concentration of AgNpP according to previously-published methods [35]. The copolymer was crosslinked with –COOH and the ionized epoxy group and was loaded with Ag particles to create a matrix distribution. These hydrogels were synthesized in sterile glass plates, which were homogeneous, soft, and transparent depending on the concentration of Ag particles. Hydrogel samples showed different gradations of yellow, and the transparency decreased with increasing concentration of Ag particles. As shown Figure 2, all samples were easily removed and were sheet-shaped. All hydrogels maintained their shape and were stable during the cross-linking process. Meanwhile, the hydrogel samples could be easily inverted on the glass plates without shape changes and separation, demonstrating their excellent adhesive properties and structural conformity.

### 3.2. Swelling Study of Hydrogel

**Figure 3:**
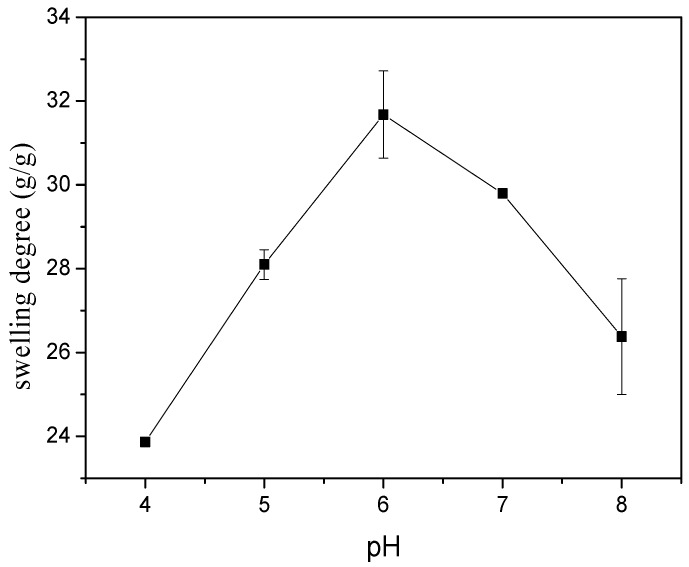
Swelling behaviors of γ-PGA hydrogel soaked in Britton–Robinson buffer solution.

After being immersed in the Britton–Robinson buffer solution for 24 h, the degree of swelling of the hydrogel film increased from 24 to 32 within a pH range of 4.0–6.0, with the pH increasing from 4.0–8.0, as shown in Figure 3. While the pH was 6.0, the swelling degree was 31.67 g/g. Since the pH value of human body fluids is close to neutral, this indicates that hydrogel films can absorb wound fluids better and prevent wound infections.

### 3.3. Diffusion Studies

Diffusion studies were conducted as a function of absorbance over time using spectroscopy. Prior to the study, the λ_max_ of AgNpP was determined. As shown in Figure 2, to carry out the study in the region of the visible spectrum, we identified 438 nm as the most appropriate wavelength for AgNpP identification.

Then, we used different concentrations corresponding to the absorbance in order to draw a standard curve (*R*^2^ = 1) and the equation for calculating the amount of leached nanoparticles was:

$$y=0.03817\;x+0.04748\;\left(R^{2}=0.9983\right)$$

**Figure 4:**
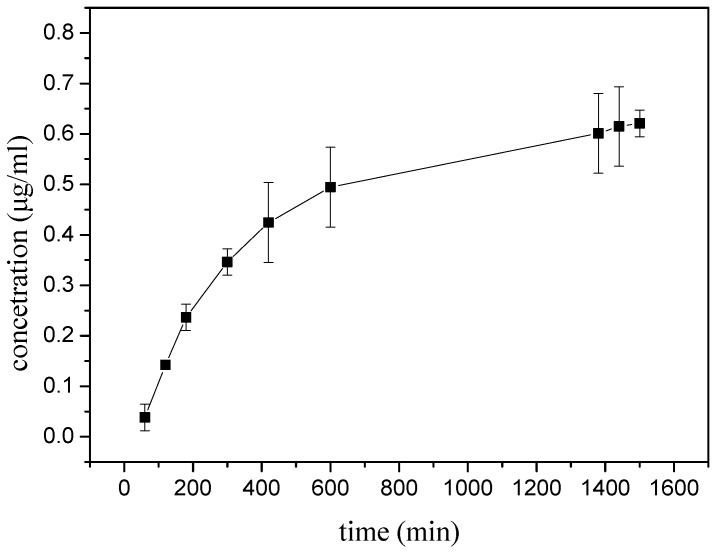
Ag particle release over time measured by UV-vis spectroscopy.

As shown in Figure 4, it was evident that the AgNpP was released from these hydrogel samples in a controlled and predictable manner. The AgNpP release study demonstrated an initial burst release, before 420 min, of sufficient quantities of Ag to create a strong adjacent antibacterial effect (which is particularly beneficial in infected wounds) [37].

Furthermore, elongation of the release duration time could increase the amount of released silver over the controlled period. This indicated that hydrogel samples could also release drugs at predictable levels. In addition, this research showed that these hydrogel systems steadily release AgNpP over two days. Some recent studies have reported that silver-load hydrogels, such as AgNPs/chitosan hydrogel [29], Ag/graphene polymer hydrogel [34], and PVA/silver nano-composite hydrogel [27], could guarantee sustained antimicrobial efficacy. The structure and condition of hydrogels will affect the size and even the electrical properties of nano-Ag. Therefore, the release process was changed. However, the Korsmeyer–Peppas model, as a release model, was widely used to describe nano-Ag release from a polymeric system. The release results indicated that our hydrogels have the potential to compete with currently-researched silver-load dressings.

### 3.4. In Vivo Study

The wound healing ability of the Ag/γ-PGA hydrogel dressing was investigated, in vivo, using Balb/C mice (Table 1). After treating with γ-PGA, γ-PGA/Ag-5, γ-PGA/Ag-20, and the control dressing, all wound closureswereobservedfor14 days, as shown in Table 1 of the wound healing process. On the first day of surgery, all groups showed no visible differences in wounds. The wounds treated with γ-PGA/Ag and γ-PGA healed faster than the control. With the increase in the concentration of silver, the wounds recovered faster. On day 14, the wounds treated with γ-PGA/Ag-20 showed that wound closure was nearly complete. The higher wound closure rate of the γ-PGA/Ag dressing was due to the synergistic effect of γ-PGA and Ag. The uniformly-adhered hydrogel absorbed the wound exudates, which could reduce the risk of dehydration that was found in the control [38]. At the same time, the hydrogel dressings could be easily removed without damaging the regenerated tissue. However, there was significant adhesion and damage to wounds when removing the gauze.

**Figure 5:**
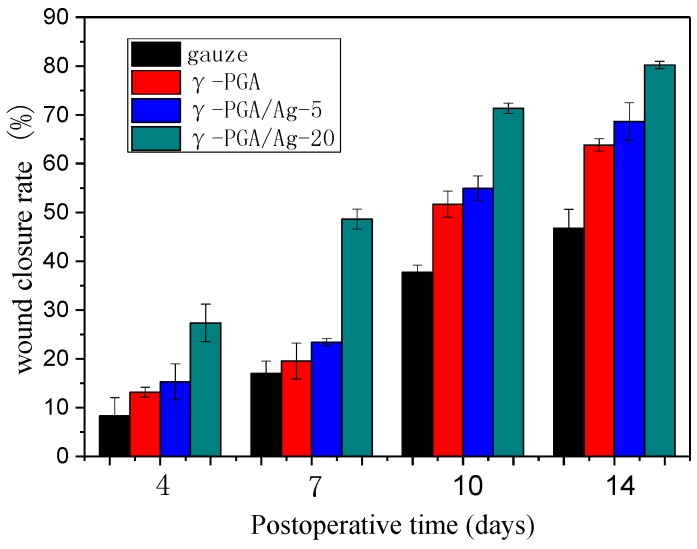
Evaluation of the wound area closure of wounds treated with neat γ-PGA, different γ-PGA/Ag, and the control.

Percentage of the wound healing rate was used to determine the wound healing ability of the tested dressings. Figure 5 shows the wound healing rates of the dressing-treated groups overtime. The wound healing rates of the gauze-treated group were 8.28 ± 3.72, 16.98 ± 2.54, 37.77 ± 1.43, 46.79 ± 3.84 at 4, 7, 10 and 14 days, respectively. The wound healing rates of the γ-PGA treated groups were 13.15 ± 0.99, 19.56 ± 3.66, 51.67 ± 2.71, 63.84 ± 1.28 at 4, 7, 10 and 14 days, respectively. The wound healing rates of the γ-PGA/Ag-5 treated groups were 15.28 ± 3.65, 23.39 ± 0.72, 54.95 ± 2.54, 68.63 ± 3.84 for 4, 7, 10 and 14 days, respectively. The wound healing rates of the γ-PGA/Ag-20 treated groups were 27.36 ± 3.85, 48.61 ± 2.06, 71.33 ± 1.09, 80.21 ± 0.72 at 4, 7, 10 and 14 days, respectively. As shown in Figure 5, the wound healing rate of the γ-PGA/Ag-20 hydrogel dressing was enhanced prominently at day 7 compared to the gauze dressing group. The excellent wound healing efficacy of the γ-PGA/Ag-20 hydrogel dressing could be due to the synergistic effects between the antibacterial ability of silver and the 3D structure of the γ-PGA hydrogel. Meanwhile, the gauze almost showed no increase in wound healing rate on days 10 and 14 (Figure 5). Therefore, the wound healing rate was greatly enhanced by the addition of silver. Furthermore, wounds healed under the γ-PGA/Ag-20 hydrogel dressing demonstrated better tissue quality with less scarring. The healing rate of chitosan/silver wound dressing was 90.98 ± 6.09 within 15 day [29]. Shi et al. [39] prepared composite hydrogel dressing with γ-PGA and silk-sericin. On the day 15, the wounds treated with the hydrogels had almost completely healed.

### 3.5. Histological Analysis

The wound tissues were H&E stained and their morphological features during the healing process were observed using a light microscope at days 4, 7, 10 and 14 (Table 2). After wounding, at day 1, biopsies from all four groups did not show a clear difference. At day 4, compared with the gauze, all hydrogel dressing worked well to block inflammatory cell infiltration. Infiltration with a marked number of inflammatory cells and defects in the epithelium appeared more commonly in the gauze group. In the tissue, bacterial growth was observed in gauze group.

As the silver concentration increased in the γ-PGA/Ag hydrogel wound dressing, inflammatory cells and bacterial colonies were decreased. However, in the γ-PGA and gauze groups, suppression was not well shown. The results indicated that silver acts in a key role in the decrease of inflammatory reaction. In the hydrogel dressing groups, the initially thin granulation tissue was poor in fibroblasts, microvessels and re-epithelialization lining. This indicated that the degree of wound remodeling was comparable to other different regimens applied to the mice. In addition, in the γ-PGA/Ag-20 hydrogel wound dressing group, inflammatory cells were remarkably reduced.

As shown in Table 2, re-epithelialization was activated on day 7 by in γ-PGA/Ag wound dressing. However, the epidermis of wounds treated using gauze and γ-PGA was unable to be activated. In addition, fat granules, fibroblasts, moderate deposition of collagen fibers, and keratin formation were more integrated in wounds treated with γ-PGA/Ag than those in the γ-PGA and gauze groups. It was shown that wounds re-epithelialized more rapidly under moist environments with antimicrobial factors [15,40]. Due to the strong adsorption capability and good tissue affinity, the γ-PGA/Ag hydrogel created a suitable condition for epithelial cell proliferation. The extent of epithelialization of a wound was improved with an increase in silver concentration in the γ-PGA/Ag. These studies indicated that γ-PGA/Ag hydrogel could create a suitable condition for wound healing, and reduce the risk of dehydration by maintaining moisture.

As shown in Table 2, histological experiments of wounds treated with γ-PGA/Ag exhibited that tissue regenerated very well, particularly in the γ-PGA/Ag-20 group on day 10; moreover, new hair follicles grew. The results from the in vivo study indicated that γ-PGA/Ag can be a potential material for wound healing.

## 4. Conclusions

A novel silver nanoparticles/poly(gamma-glutamic acid) (γ-PGA) composite dressing was successfully synthesized using a simple method for biomedical applications, such as wound healing. The γ-PGA/Ag dressing showed high water absorption, which could enhance the moisture retention time and is helpful in accelerating wound healing.

In vivo wound healing experiments showed that the γ-PGA/Ag hydrogel could enhance wound healing compared with γ-PGA hydrogel and gauze. It also indicated excellent re-epithelialization and dense collagen deposition and a reduction in inflammatory cells. The greatest Ag release was also found in the γ-PGA/Ag-5 hydrogel dressing. Overall, these results demonstrated that the γ-PGA/Ag dressing has the potential to be applied in wound healing. We will also investigate chronic and burning wound healing in future work.

## Figures and Tables

**Table 1:** In vivo study on the effects of the treatment of wound infections for mice with gauze and different γ-PGA/Ag PEC s hydrogels.

A	B	Day 0	Day 4	Day 7	Day 10	Day 14
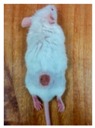	gauze	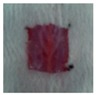	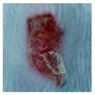	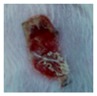	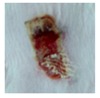	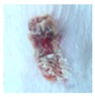
	γ-PGA	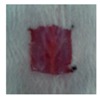	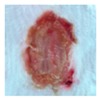	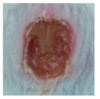	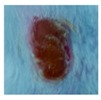	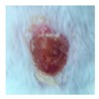
↓	γ-PGA/Ag-5	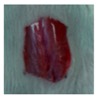	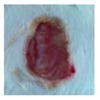	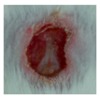	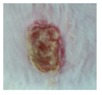	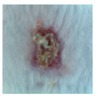
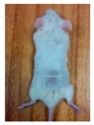						
	γ-PGA/Ag-20	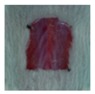	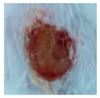	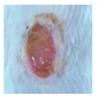	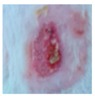	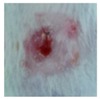

**Table 2:** Histopathological evaluation of skin sections. Micrographs of H&E stained wounds treated with gauze and different γ-PGA/Ag dressings at different time intervals (4, 7, 10, and 14 days) (magnification 100×).

Wound Dressing	Day 4	Day 7	Day 10	Day 14
gauze	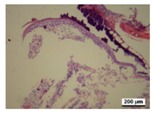	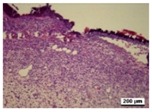	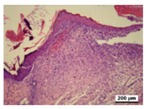	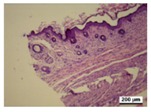
γ-PGA	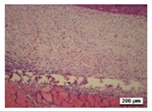	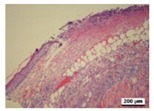	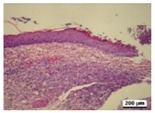	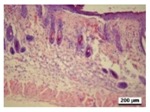
γ-PGA/Ag-5	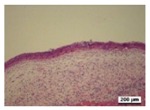	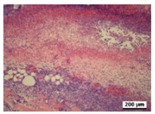	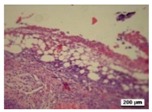	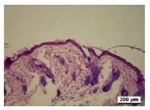
γ-PGA/Ag-20	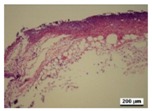	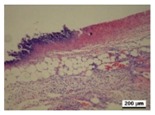	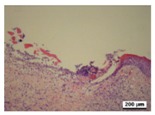	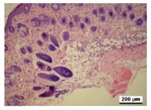
Positivecontrol